# A Conversation with Loreto Paulino Jr.

**DOI:** 10.1021/acscentsci.4c00192

**Published:** 2024-02-15

**Authors:** Robin Meadows

In the summer of 2023, on the heels of graduating from the University of Guam with his bachelor’s in chemistry, Loreto Paulino Jr. set up camp in Alaska. He was there as part of the Polaris Project, which brings young scientists on climate change-related research expeditions in the Arctic. He and other project participants were temporarily stationed in the Yukon-Kuskokwim Delta, a vast tundra on the Bering Sea.

**Figure:**
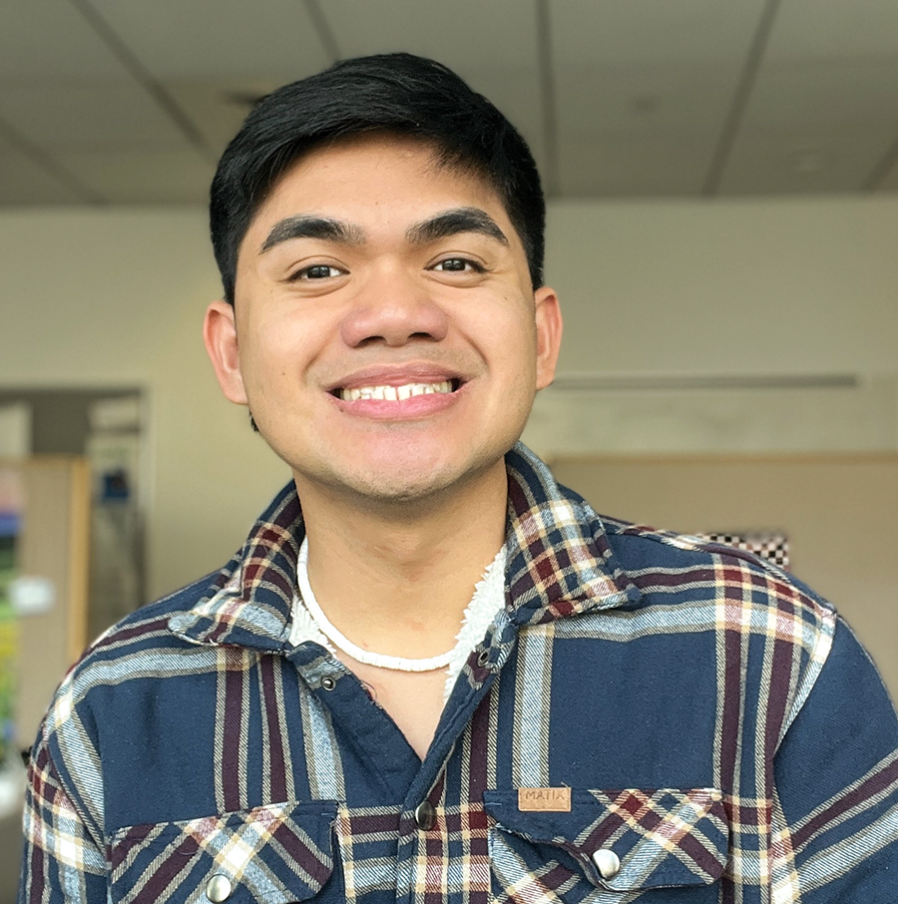
Photo courtesy of Loreto Paulino Jr.

This remote site lacks roads, so the crew assembled in the town of Bethel, Alaska, and traveled the 95 km to base camp by float plane. For the next 2 weeks, Paulino lived in a tent and made the daily trek to and from his study site—though he occasionally got a lift in a helicopter.

Before the expedition embarked, program director Susan Natali mentioned that none of the Polaris Project students had studied beavers. Paulino jumped at the chance. Beavers have long lived in Alaska’s forests, but the tundra to the north has become so warm due to climate change that the animals have invaded it. It’s important to understand how their presence there may affect this fragile ecosystem. For example, beaver ponds may let nutrient-laden sediments collect rather than wash downstream and can favor anaerobic bacteria that produce the greenhouse gases carbon dioxide and methane.

Paulino studied the impact of beavers on nutrients and greenhouse gases in the tundra and presented his work at the fall 2023 meeting of the American Geophysical Union.

Robin Meadows talked with Paulino about his field experience in the Arctic and how it shaped him as a researcher, as well as how being a Pacific Islander brings challenges and urgency to his research. This interview was edited for length and clarity.

## What was your project, and how did the experience help you grow as a researcher?

Beavers are a hot topic in the Arctic because in other places they change the landscape a lot.

The question is, how is the Arctic impacted by beavers? My assumption was that beaver dams would affect the concentration of nutrients in the water. I sampled water at 21 sites with different beaver activity levels. People always ask if I saw beavers, but I didn’t. I saw lodges, dams, and gnawed branches that were evidence of activity.

I tested water upstream and downstream of dams for nitrogen, phosphorus, and carbon. While I found no significant differences, further research with larger sample sizes might show differences. I also took gas samples with bubble traps—a syringe attached to a funnel on the water surface—to measure nitrogen, carbon dioxide, and methane releases into the atmosphere, and I’m still waiting to analyze them.

I worked on mathematical models of coral recovery at the University of Guam and am now developing mathematical models of carbon sequestration by marine microbes in the deep ocean at Boston University. As a researcher, I now believe that I can conduct my own research projects both computationally and in the field. If my future projects need further data for a model, I’ll just go grab it myself.

## What was the most challenging part of your project?

My field site was about 2 km from base camp. That doesn’t seem like much, but on the arctic terrain, 1 km feels like 4 km. The peatland is marshy so when you step on it, your feet get stuck. It took about an hour each way. Walking back to camp was quite dreadful. It was great that the sun hardly went down because some days I didn’t get back until around 7 p.m. Because it was summer it was relatively warm in Alaska, but coming from Guam, it felt quite cold to me. And there were a lot of mosquitoes.

**Figure:**
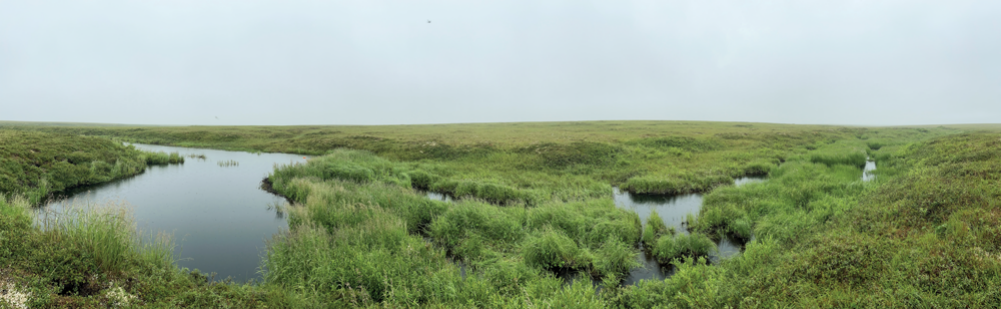
Beaver dams made Paulino’s study sites lusher than the rest of the tundra in the Yukon-Kuskokwim Delta, Alaska. Credit: Gabriel Duran.

## What did you enjoy most?

My favorite part was the sites where the beavers were located. Compared to all the sites I have seen in the Arctic, those areas are beautiful. Everywhere else is kind of barren.

Near beaver dams, you see flowers and plants that you don’t really see anywhere else around that terrain. The sounds of the water exiting the dams rang with a sense of tranquility. The sites make you forget how harsh the conditions can get in the Arctic. It’s quite amazing—the level of change these beavers can achieve by just being present in a landscape. Beavers are known to change their environment, but seeing it firsthand, the level of change was amazing.

## What is your dream career−where do you want to be in 20 years?

I see myself as a chemistry professor, doing research in chemical oceanography. One group of compounds I am interested in is recalcitrant dissolved organic carbon [RDOC], which can persist in the deep ocean for long periods. RDOC has potential for carbon sequestration, which can help with climate change, but little is known about mechanisms of its production by marine bacteria.

I’ve been interested in DOC since I was an undergrad, and one of my projects as a research fellow at Boston University is looking into a marine bacterial strain database and using genome modeling to investigate relevant metabolites. I feel DOC in the ocean is where my research focus will head in the future.

**Figure:**
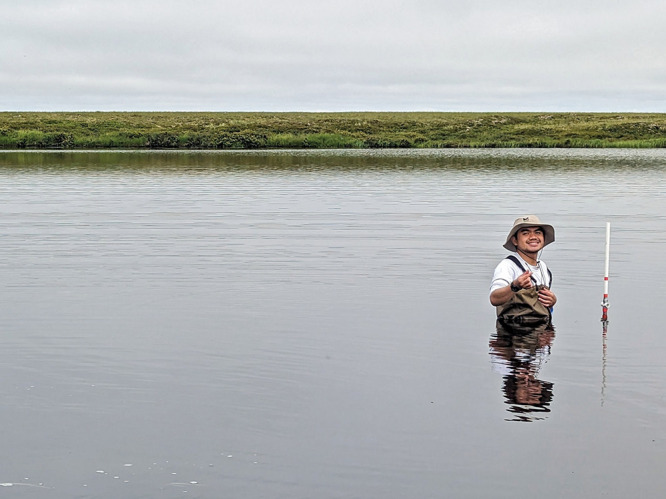
Loreto Paulino Jr. installing a bubble trap to collect gases released at a study site in the Yukon-Kuskokwim Delta, Alaska. Credit: Nigel Golden.

## As a Pacific Islander, have you experienced challenges or barriers to becoming a scientist?

On my journey as a scientist, although most of it has been positive, I have faced challenges and barriers as a Pacific Islander. In conferences and academic programs, I have directly experienced stereotypical attitudes about Pacific Islanders, such as the infamous, “How do you speak English so well?”

In the same setting, I have also indirectly heard comments that the University of Guam does not provide education of the same quality as mainland schools. I found this challenging as I sometimes felt it hard to communicate, fearing there was some preconceived negative idea or ignorance about the school. This is compounded by Pacific Islanders’ lack of representation in all the settings I’m in outside Guam.

As a scientist, I find not only diversity but inclusion and equity important in STEM. A diverse community is significant only if everyone feels included in the discussion, and it also means nothing if not everyone is given the same resources to succeed. Diversity in STEM is essential as it fosters representation from different backgrounds, allowing a sense of voice in crucial discussions.

## How does it feel to do climate research as someone from Guam, a place that faces climate threats?

Guam is really affected by sea level rise. But we don’t have a voice: we don’t even get to vote, can’t present bills, and have no say in who our president is. Pacific Islanders are less than 1% of STEM, and it’s an honor to represent the next generation of Pacific Islanders so they can be part of the conversation. Giving them that voice is really important to me.

## Robin Meadows is a freelance contributor to

Chemical & Engineering News, *the independent news outlet of the American Chemical Society.*

